# Efficacy of polyethylene glycol loxenatide for type 2 diabetes mellitus patients: a systematic review and meta-analysis

**DOI:** 10.3389/fphar.2024.1235639

**Published:** 2024-02-26

**Authors:** Yibo Liu, Wenjing Ma, Hui Fu, Zhe Zhang, Yanyan Yin, Yongchun Wang, Wei Liu, Shaohong Yu, Zhongwen Zhang

**Affiliations:** ^1^ Department of Endocrinology and Metabology, Rehabilitation Hospital, The Second Affiliated Hospital of Shandong University of Traditional Chinese Medicine, The Third Affiliated Hospital of Shandong First Medical University, Jinan, China; ^2^ Rehabilitation Hospital, The Second Affiliated Hospital of Shandong University of Traditional Chinese Medicine, Jinan, China; ^3^ Medical Integration and Practice Center, Shandong University, Jinan, China; ^4^ Shandong University of Traditional Chinese Medicine, Jinan, China; ^5^ Shandong Provincial Medical Association, Jinan, China; ^6^ Teaching and Research Section of Internal Medicine, College of Medicine, Shandong University of Traditional Chinese Medicine, Jinan, China; ^7^ Shandong Provincial Key Laboratory for Rheumatic Disease and Translational Medicine, Department of Endocrinology and Metabology, The First Affiliated Hospital of Shandong First Medical University and Shandong Provincial Qianfoshan Hospital, The Third Affiliated Hospital of Shandong First Medical University, Jinan, China

**Keywords:** polyethylene glycol loxenatide, type 2 diabetes mellitus, blood glucose, blood lipid profiles, blood pressure, body mass index, body weight, meta-analysis

## Abstract

**Objective:** Some studies have proved that polyethylene glycol loxenatide (PEG-Loxe) has significant effects on controlling blood glucose and body weight in patients with type 2 diabetes mellitus (T2DM), but there is still some controversy over the improvement of blood lipid profiles (BLP) and blood pressure (BP), and more evidences are needed to verify such effects. Therefore, this study was conducted to provide a comprehensive evaluation of the efficacy of PEG-Loxe in improving blood glucose (BG), BLP, BP, body mass index (BMI), and body weight (BW) in patients with T2DM for clinical reference.

**Methods:** Randomized controlled trials (RCT) in which PEG-Loxe was applied to treat T2DM were retrieved by searching PubMed, Cochrane Library, Embase, Medline, Scopus, Web of Science, China National Knowledge Infrastructure, China Scientific Journal, Wanfang Data, and SinoMed databases. Outcome measures included BG, BLP, BP, BMI, and BW. RevMan 5.3 software was used to perform data analysis.

**Results:** Eighteen trials were identified involving 2,166 patients. In experimental group 1,260 patients received PEG-Loxe alone or with other hypoglycemic agents, while in control group 906 patients received placebo or other hypoglycemic agents. In the overall analysis, PEG-Loxe significantly reduced the levels of glycosylated hemoglobin (HbA1c), fasting plasma glucose (FPG), 2-h postprandial blood glucose (2-h PBG), BMI, and BW compared with control group. However, it had no obvious effect on total cholesterol (TC), triglycerides (TG), low-density lipoprotein cholesterol (LDL-C), high-density lipoprotein cholesterol (HDL-C), systolic blood pressure (SBP), and diastolic blood pressure (DBP).

**Conclusion:** PEG-Loxe has better hypoglycemic effects compared with placebo in patients with T2DM, but could not significantly improved TG, LDL-C, HDL-C, SBP, and DBP. And the combination of conventional hypoglycemic drugs (CHD) and PEG-Loxe could more effectively improve the levels of HbA1c, FPG, 2-h PBG, TC, TG, BMI, and BW compared with CHD in T2DM patients.

**Systematic Review Registration:** www.inplasy.com, identifier INPLASY202350106

## 1 Introduction

Diabetes is one of the most serious and long-term chronic diseases and is also one of the top 10 causes of death in adults. Therefore, it poses a major threat to individual, family and global health (Saeedi et al., 2019). According to the International Diabetes Federation, more than 500 million individuals suffered from diabetes in 2021 worldwide, and it is expected that the number of patients will increase by 200 million in 2045. In 2021, the global health costs associated with diabetes were evaluated at 966 billion U.S. dollars, and this number is expected to reach 1,054 billion U.S. dollars by 2045 (Williams et al., 2020; Sun et al., 2022). With the aging of the global population and changes in lifestyle, there would be more people suffering from diabetes and more cost spending diabetes. Type 2 diabetes mellitus (T2DM), the most prevalent diabetes, accounts for more than 90% diabetic patients (Zheng et al., 2018; Khan et al., 2019). T2DM is a metabolic disease induced by a variety of causes. It would lead to insulin deficiency, insulin resistance, and persistently elevated blood glucose levels. In a long-term hyperglycemic internal environment, blood vessels and nerves would undergo pathological changes, which could damage the organs such as heart, kidneys, and eyes (Chatterjee et al., 2017; Ahmad et al., 2022). Since there is no radical cure for T2DM at present, blood glucose and weight control are particularly critical in its treatment process (Davies et al., 2022).

In recent years, since glucagon-like peptide-1 receptor agonists (GLP-1RAs) have significant hypoglycemic effects and multiple benefits for diabetic patients, they have been recommended in major guidelines. GLP-1RAs are potent hypoglycemic agents with the function to promote glucose-dependent insulin secretion from pancreatic beta-cells by binding to glucagon-like peptide-1 receptor (GLP-1R) and inhibiting glucagon secretion (Drucker, 2018). The degradation and destroy of GLP-1RAs are slow, so the effect of reducing blood glucose (BG) could be maintained for a long time (Chen et al., 2017). In addition, GLP-1RAs have the advantages of reducing BG without increasing the incidence of hypoglycemia (Drucker and Nauck, 2006). Therefore, the 2020 American Association of Clinical Endocrinologists guidelines recommend GLP-1RAs as the drug of choice after metformin (Garber et al., 2020). Polyethylene glycol loxenatide (PEG-Loxe), a new agent of the GLP-1RAs, was approved for clinical application in China In 2019. It was synthesized by replacing the chemical structure of exenatide at the N-terminal positions 2, 14, 28, and 39, and modified by polyethylene glycol (PEG). PEG-Loxe could further resist the rapid degradation of dipeptidyl peptidase-IV (DPP-4), reduce the toxicity and its antigenic immunity, prolong the mean half-life (131.8–139.8 h) and duration of action, and improve its bioavailability, compliance, and the therapeutic effect in the body, with better effects compared with exenatide (Yang et al., 2015; Chen et al., 2017). In terms of the hypoglycemic effect, studies have reported that PEG-Loxe is likely to inhibit β-cell apoptosis to promote the expression of GLP-1R, thereby activating the insulin PI3K/AKT pathway, promoting insulin synthesis and secretion, and thus exerting a hypoglycemic effect (Zhang et al., 2021). PEG-Loxe has shown a good effect on controlling BG in patients with T2DM, but there are still some controversy over the improvement of BLP and few clinical evidence for reducing BW. Therefore, we aimed to comprehensively evaluate the efficacy of PEG-Loxe for BG, BLP, BP, body mass index (BMI), and BW.

## 2 Materials and methods

The protocol and report of this study followed the “Preferred Reporting Items for Systematic Reviews and Meta-Analyses (PRISMA)” statement (Page et al., 2021) and were registered in the INPLASYInternational Platform of Registered Systematic Review and Meta-analysis Protocols (identifier: INPLASY202350106. DOI number: 10.37766/inplasy 2023.5.0106).

### 2.1 Literature search strategies

Literature was retrieved in the PubMed, Cochrane Library, Embase, Medline, Scopus, Web of Science, China National Knowledge Infrastructure (CNKI), China Scientific Journal, Wanfang Data, and SinoMed databases. The search terms were “polyethylene glycol loxenatide” or “PEG-Loxe” or “PEX168” in combination with “randomized controlled trial,” “randomized controlled trials” “RCT,” “RCTs,” “type 2 diabetes mellitus” or “diabetes mellitus” or “diabetes mellitus, type 2,” or “T2DM.” The complete search strategies of databases were shown in Supplementary Table S1.

### 2.2 The inclusion and exclusion criteria

The inclusion criteria followed the PICOS principle. T2DM patients with FPG≥11.1 mmol/L, HbA1c ≥ 9.0%; BMI≥27 kg/m^2^, age≥18 years old; Patients in experimental group received PEG-Loxe alone or along with other hypoglycemic agents, and patients in control group received placebo or other hypoglycemic agents; The dose of PEG-Loxe was 0.1 mg or 0.2 mg. Outcome indicators involved HbA1c, FPG, 2-h PBG, TC, TG, LDL-C, HDL-C, SBP, DBP, BMI and BW; RCT published in English or Chinese.

The exclusion criteria were shown as follows: the study design was scientific research achievements, systematic reviews, and animal experiments; trials that did not report related information; the full text could not be obtained; other intervention measures existed; patients that combined with other severe diseases or limb dysfunction, and serious complications of T2DM.

### 2.3 Quality assessment and data extraction

The quality assessment and data extraction were conducted by 2 researchers independently, with disagreements resolved by consensus. The quality of the included studies was assessed according to six aspects: random sequence generation (selection bias), allocation concealment (selection bias), blinding of participants and personnel (performance bias), blinding of outcome assessment (detection bias), incomplete outcome data (attrition bias), and selective reporting (reporting bias), which are detailed described in the Cochrane Collaboration Risk-of-Bias Tool (Higgins et al., 2011). Information extracted from each study included the first author, year of publication, sample size, age range, intervention measures, duration, and outcomes.

### 2.4 Statistical analysis

RevMan 5.3 software was used for data analysis. Mean difference (MD) and 95% confidence intervals (CI) were used to represent continuous variables. *p* < 0.05 was considered statistically significant. The statistical heterogeneity was evaluated by Chi-square and I^2^ tests. According to section-10–10-four to one of Cochrane Handbook for Systematic Reviews of Intervention, the confidence interval of estimate around the random effects was wider than the fixed effects when heterogeneity was present. Therefore, results of non-heterogeneous (I^2^<50%) and heterogeneous (I^2^˃50%) were analyzed by fixed or random effects models for calculating the pooled effect, respectively (Cumpston et al., 2019). Subgroup analysis was performed based on different intervention measures, dosages and treatment time. The experimental group was divided into PEG-Loxe combined with conventional hypoglycemic drugs (PEG-Loxe + CHD) group and PEG-Loxe group, while the control group was divided into CHD group, CHD combined with placebo (CHD + Placebo) group and Placebo group. PEG-Loxe group was further divided into 0.1 mg and 0.2 mg subgroup. And treatment courses were divided into 12 and 24 weeks. In addition, sensitivity analysis was executed when statistically significant heterogeneity was observed (Patsopoulos et al., 2008; Ruppar, 2020).

## 3 Results

### 3.1 Study selection and characteristics

**FIGURE 1:**
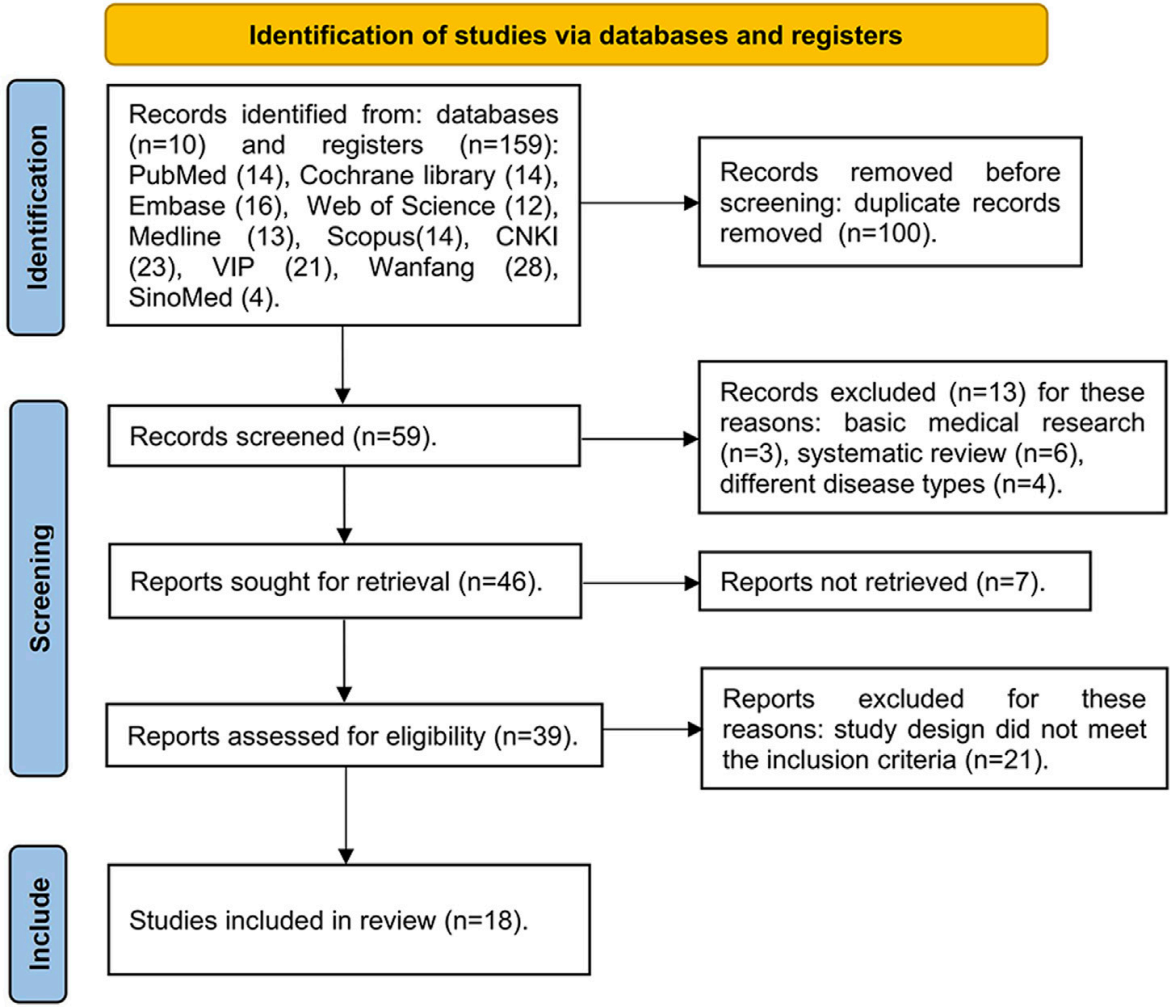
The flowchart of literature search.

**FIGURE 2:**
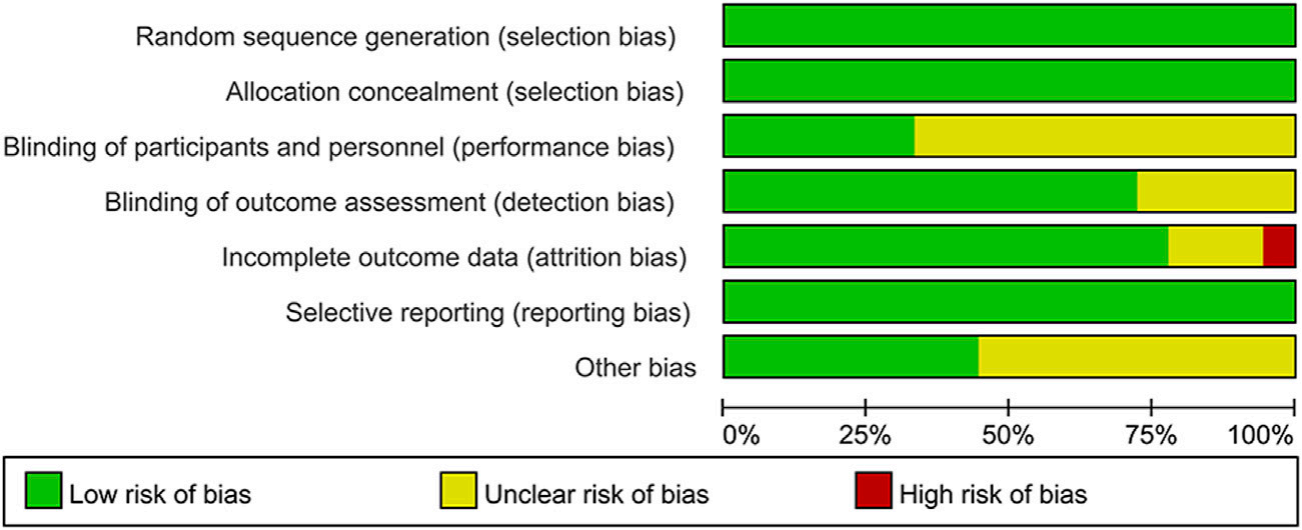
Risk of bias graph.

One hundred and fifty-nine relevant articles were retrieved, 59 articles were obtained after eliminating duplicate articles, 46 articles were screened after reading the titles and abstracts, and finally 18 articles (Chen et al., 2017; Yao et al., 2017; Gao et al., 2020; Li et al., 2021; Liang et al., 2021; Shuai et al., 2021; Wang and Zhao, 2021; Li K. et al., 2022; Li X. Y. et al., 2022; Tian et al., 2022; Yang, 2022; Zhao et al., 2022; Song et al., 2023; Wan et al., 2023; Zhang S. et al., 2023; Zhang Y. et al., 2023; Zhong, 2023; Zhou et al., 2023) were included after full-text reading, involving 2,166 patients in total (experimental group: 1,260 patients; control group: 906 patients). The literature search process is shown in Figure 1. Table 1 presents the basic information of these articles. The risk of bias assessments of the studies are showed in Figure 2.

**TABLE 1:** The characteristics of the included studies.

ID	Group	Sample size	Mean age, years	Intervention measures	Treatment time, weeks	Outcome indicator
Yang (2022)	Experimental group	40	55.89 ± 2.52	PEG-Loxe 0.1 mg	12	HbAlc, FPG, and 2-h PBG
	Control group	40	55.82 ± 2.56	Insulin glargine		
Wan et al. (2023)	Experimental group	35	63.31 ± 6.43	PEG-Loxe 0.2 mg + Insulin glargine + Metformin	12	HbAlc, FPG, TC, TG, LDL-C, and BMI
	Control group	35	63.25 ± 6.36	Insulin glargine + Metformin		
Zhang Y. et al. (2023)	Experimental group	41	53.50 ± 5.43	PEG-Loxe 0.2 mg + Metformin	12	HbA1c, FBG, 2-h PBG, TC, TG, LDL-C, HDL-C
	Control group	41	53.00 ± 5.45	Metformin		
Zhong (2023)	Experimental group	30	53.30 ± 10.86	PEG-Loxe 0.2 mg + Insulin glargine	12	HbA1c, FBG, 2-h PBG, TC, TG
	Control group	30	52.50 ± 10.88	Insulin glargine		
Zhou et al. (2023)	Experimental group	40	46.8 ± 11.3	PEG-Loxe 0.2 mg + Metformin	12	HbA1c, FBG, 2-h PBG, TC, TG, LDL-C, HDL-C, SBP, DBP, and BMI
	Control group	40	47.2 ± 12.1	Metformin		
Li et al. (2021)	Experimental group	30	47.7 ± 6.8	PEG-Loxe 0.1 mg + Metformin/Acarbose	12	HbAlc, FPG, 2-h PBG, and BMI
	Control group	30	47.2 ± 7.3	Metformin/Acarbose		
Tian et al. (2022)	Experimental group	35	50 ± 13.00	PEG-Loxe 0.2 mg + Metformin	12	HbA1c, FBG, 2-h PBG, BMI, and BW
	Control group	34	50 ± 13.00	Sodium chloride injection + Metformin		
Zhao et al. (2022)	Experimental group	56	46.72 ± 9.34	PEG-Loxe 0.2 mg + Metformin	12	HbA1c, FBG, 2-h PBG, TC, TG, LDL-C, HDL-C, and BMI
	Control group	54	47.89 ± 8.95	Metformin		
Li K. et al. (2022)	Experimental group	50	52.34 ± 4.15	PEG-Loxe 0.2 mg + Metformin	12	FPG, 2-h PBG, TG, TC, LDL-C, and HDL-C
	Control group	50	52.56 ± 4.08	Metformin		
Liang et al. (2021)	Experimental group	62	53.8 ± 8.5	PEG-Loxe 0.2 mg	24	HbAlc, FPG, 2-h PBG, TG, TC, HDL-C, LDL-C, and BMI
	Control group	62	54.1 ± 7.9	Multiple oral hypoglycemic drugs or oral drugs combined with insulin		
Wang and Zhao (2021)	Experimental group	37	59.78 ± 14.76	PEG-Loxe 0.2 mg	12	HbAlc, FPG, 2-h PBG, and BMI
	Control group	37	58.36 ± 14.63	Insulin glargine		
Yao et al. (2017)	Experimental group	13	53.6 ± 9.90	PEG-Loxe 0.1 mg	12	HbAlc, FPG, and 2-h PBG
		12	53.6 ± 9.90	PEG-Loxe 0.2 mg		
	Control group	11	53.6 ± 9.90	Placebo		
Gao et al. (2020)	Experimental group	179	53.60 ± 10.50	PEG-Loxe 0.1 mg + Metformin	24	HbA1c, FPG, 2-h PBG, TC, TG, HDL-C, LDL-C, SBP, DBP, and BW
		175	52.80 ± 10.60	PEG-Loxe 0.2 mg + Metformin		
	Control group	179	52.30 ± 10.70	Placebo + Metformin		
Shuai et al. (2020)	Experimental group	124	50.50 ± 10.40	PEG-Loxe 0.1 mg	24	HbA1c, FPG, 2-h PBG, TC, TG, HDL-C, LDL-C, SBP, DBP, and BW
		116	52.40 ± 11.50	PEG-Loxe 0.2 mg		
	Control group	121	51.50 ± 10.90	Placebo		
Chen et al. (2017)	Experimental group	41	52.60 ± 8.40	PEG-Loxe 0.1 mg + Metformin	12	HbA1c, FPG, 2-h PBG, TC, TG, HDL-C, LDL-C, SBP, DBP, and BW
		39	49.80 ± 10.90	PEG-Loxe 0.2 mg + Metformin		
	Control group	38	53.50 ± 10.20	Placebo + Metformin		
Zhang S. et al. (2023)	Experimental group	35	68.30 ± 10.40	PEG-Loxe 0.2 mg + Metformin	24	HbA1c
	Control group	34	67.40 ± 10.20	Insulin glargine + Metformin		
Song et al. (2023)	Experimental group	50	51.38 ± 6.39	PEG-Loxe 0.2 mg + Metformin + Insulin	24	HbA1c, FPG, 2-h PBG, TC,TG, HDL-C, LDL-C, BW, and BMI
	Control group	50	51.49 ± 6.67	Metformin + Insulin		
Li X. Y. et al. (2022)	Experimental group	20	63.29 ± 1.27	PEG-Loxe 0.2 mg + Metformin	12	FPG, 2-h PBG, TG, HDL-C, and LDL-C
	Control group	20	64.23 ± 1.31	Metformin		

PEG-Loxe, polyethylene glycol loxenatide; HbA1c: glycosylated hemoglobin; FPG, fasting plasma glucose; 2-h PBG, 2-h postprandial blood glucose; TC, total cholesterol; TG, triglycerides; LDL-C, low-density lipoprotein cholesterol; HDL-C, high-density lipoprotein cholesterol; SBP, systolic blood pressure; DBP, diastolic blood pressure; BMI, body mass index; BW, body weight.

### 3.2 The results of meta-analyses

The results of overall and subgroup analysis are present in Tables 2, 3, respectively.

**TABLE 2:** The results of overall analysis.

Index	Dosage (mg)	n	Sample size	Effect model	Overall effect: Heterogeneity (P_h_, I^2^%); MD (95% CI); Z-test (Z-values, P_Z_)
HbA1c	0.1	6	846	Random	P_h_ < 0.00001, 98%; −0.91 (−1.05, −0.76); 12.16, P_Z_ < 0.00001
	0.2	14	1,169		
FPG	0.1	7	886	Random	P_h_ < 0.00001, 97%; −1.22 (−1.42, −1.02); 12.06, P_Z_ < 0.00001
	0.2	14	1,545		
2-h PBG	0.1	7	886	Random	P_h_ < 0.00001, 97%; −1.84 (−2.16, −1.53); 11.37, P_Z_ < 0.00001
	0.2	13	1,475		
TC	0.1	3	682	Random	P_h_ < 0.00001, 93%; −0.44 (−0.68, 0.19); 3.48, P_Z_ = 0.0005
	0.2	11	1,384		
TG	0.1	3	682	Random	P_h_ < 0.00001, 97%; −0.59 (−0.98, 0.19); 2.92, P_Z_ = 0.004
	0.2	11	1,384		
LDL-C	0.1	4	722	Random	P_h_ < 0.00001, 93%; −0.16 (−0.34, 0.02); 1.79, P_Z_ = 0.07
	0.2	10	1,324		
HDL-C	0.1	3	682	Random	P_h_ < 0.00001, 88%; 0.07 (−0.01, 0.14); 1.77, P_Z_ = 0.08
	0.2	9	1,254		
SBP	0.1	3	344	Random	P_h_ < 0.00001, 93%; 0.17 (−0.90, 1.24); 0.31, P_Z_ = 0.75
	0.2	4	370		
DBP	0.1	3	344	Random	P_h_ < 0.00001, 95%; −0.39 (−1.20, 0.42); 0.95, Pz = 0.34
	0.2	4	370		
BMI	0.1	2	418	Random	P_h_ < 0.00001, 82%; −1.68 (−2.20, −1.17); 6.44, P_Z_ < 0.00001
	0.2	9	1,048		
BW	0.1	2	603	Random	P_h_ < 0.00001, 96%; −2.71 (−4.97, −0.45); 2.35, P_Z_ = 0.02
	0.2	5	870		

P_h_, *p*-values for heterogeneity of Q-test; MD, mean difference; CI, confidence interval; Pz, *p*-values for Z-test; P_Z_ < 0.05, shows a significant difference; HbA1c, glycosylated hemoglobin; FPG, fasting plasma glucose; 2-h PBG, 2-h postprandial blood glucose; TC, total cholesterol; TG, triglycerides; LDL-C, low-density lipoprotein cholesterol; HDL-C, high-density lipoprotein cholesterol; SBP, systolic blood pressure; DBP, diastolic blood pressure; BMI, body mass index; and BW, body weight.

**TABLE 3:** The results of subgroup analysis.

Index	Heterogeneity (P_h_, I^2^%); MD (95% CI); Z-test (Z-values, P_Z_)							
	Intervention measure subgroup				Dosage subgroup		Treatment time subgroup	
	PEG-Loxe + CHD		PEG-Loxe					
	CHD	CHD + Placebo	CHD	Placebo	0.1 mg	0.2 mg	12 weeks	24 weeks
HbA1c	P_h_ < 0.00001, 96%	P_h_ = 0.008, 75%	P_h_ = 0.06, 71%	P_h_ < 0.00001, 99%	P_h_ = 0.03, 60%	P_h_ < 0.00001, 99%	P_h_ < 0.00001, 90%	P_h_ < 0.00001, 100%
	−1.04 (−1.38, −0.69)	−0.81 (−0.84, −0.77)	−0.72 (−2.30, 0.86)	−1.05 (−1.34, −0.76)	−0.83 (−0.88, −0.79)	−1.01 (−1.35, −0.66)	−1.16 (−1.57, −0.75)	−0.59 (−0.89, −0.28)
	5.86, P_z_ < 0.00001	43.24, P_z_ < 0.00001	0.90, P_z_ = 0.37	7.07, P_z_ < 0.00001	39.02, P_z_ < 0.00001	12.16, P_z_ < 0.00001	5.55, P_z_ < 0.00001	3.57, P_z_ = 0.0002
FPG	P_h_ = 0.10, 37%	P_h_ < 0.00001, 100%	P_h_ = 0.0002, 93%	P_h_ < 0.00001, 97%	P_h_ < 0.00001, 96%	P_h_ < 0.00001, 87%	P_h_ = 0.03, 46%	P_h_ < 0.00001, 99%
	−1.04 (−1.25, −0.84)	−1.23 (−2.32, −0.15)	−1.21 (−3.03, 0.60)	−1.43 (−1.80, −1.06)	−1.28 (−1.58, −0.98)	−1.19 (−1.37, −1.00)	−1.10 (−1.37, −0.83)	−1.16 (−1.45, −0.87)
	10.10, P_z_ < 0.00001	2.23, P_z_ = 0.03	1.31, P_z_ = 0.19	7.52, P_z_ < 0.00001	8.38, P_z_ < 0.00001	12.62, P_z_ < 0.00001	7.94, P_z_ < 0.00001	7.74, P_z_ < 0.00001
2-h PBG	P_h_ = 0.05, 50%	P_h_ < 0.00001, 86%	P_h_ = 0.004, 88%	P_h_ < 0.00001, 99%	P_h_ < 0.0001, 80%	P_h_ < 0.00001, 97%	P_h_ < 0.0001, 69%	P_h_ < 0.00001, 99%
	−2.15 (−2.70, −1.59)	−1.59 (−1.85, −1.32)	−1.27 (−3.53, 0.99)	−2.12 (−3.35, −0.90)	−1.33 (−1.56, −1.10)	−1.91 (−2.42, −1.41)	−2.20 (−2.74, −1.66)	−1.57 (−2.04, −1.10)
	7.58, P_z_ < 0.00001	11.41, P_h_ < 0.00001	1.10, P_z_ = 0.27	3.40, P_z_ = 0.0007	11.37, P_z_ < 0.00001	7.42, P_z_ < 0.00001	7.97, P_z_ < 0.00001	6.51, P_z_ < 0.00001
TC	P_h_ < 0.00001, 87%	P_h_ < 0.0001, 87%	NA	P_h_ = 0.52, 0%	P_h_ = 0.0008, 86%	P_h_ < 0.00001, 92%	P_h_ < 0.0001, 81%	P_h_ < 0.00001, 94%
	−0.77 (−1.06, −0.48)	−0.10 (−0.44, 0.24)		0.23 (0.07, 0.40)	−0.03 (−0.41, 0.36)	−0.55 (−0.83, −0.27)	−0.71 (−1.00, −0.42)	0.09 (−0.24, 0.42)
	5.24, P_z_ < 0.00001	0.58, P_z_ = 0.56		2.78, P_z_ = 0.005	0.14, P_z_ = 0.89	3.82, P_z_ = 0.0001	4.78, P_z_ < 0.00001	0.54, P_z_ = 0.59
TG	P_h_ < 0.00001, 98%	P_h_ = 0.90, 0%	NA	P_h_ = 0.56, 0%	P_h_ = 0.18, 39%	P_h_ < 0.00001, 98%	P_h_ < 0.0001, 98%	P_h_ = 0.007, 79%
	−1.05 (−1.57, −0.52)	0.11 (−0.07, 0.30)		0.14 (−0.28, 0.56)	−0.03 (−0.31, 0.24)	−0.80 (−1.28, −0.32)	−0.89 (−1.54, −0.23)	−0.02 (−0.31, 0.28)
	3.98, P_z_ = 0.0001	1.21, P_z_ = 0.22		0.65, P_z_ = 0.52	0.24, P_z_ = 0.81	3.25, P_z_ = 0.001	2.64, P_z_ = 0.008	0.01, P_z_ = 0.92
LDL-C	P_h_ < 0.00001), 95%	P_h_ < 0.00001, 90%	NA	P_h_ = 0.51, 0%	P_h_ = 0.04, 64%	P_h_ < 0.00001, 95%	P_h_ < 0.00001, 93%	P_h_ = 0.04, 59%
	−0.27 (−0.56, 0.01)	−0.02 (−0.33, 0.29)		−0.00 (−0.14, 0.13)	0.00 (−0.21, 0.21)	−0.21 (−0.44, 0.02)	−0.34 (−0.65, 0.02)	0.13 (0.04, 0.22)
	1.87, P_z_ = 0.06	0.13, P_z_ = 0.90		0.05, P_z_ = 0.96	0.01, P_z_ = 0.99	1.79, P_z_ = 0.07	2.12, P_z_ = 0.03	2.71, P_z_ = 0.007
HDL-C	P_h_ < 0.00001, 91%	P_h_ = 0.01, 72%	NA	P_h_ = 0.63, 0%	P_h_ = 0.01, 74%	P_h_ < 0.00001, 91%	P_h_ < 0.00001, 92%	P_h_ = 0.03, 63%
	0.10 (−0.09, 0.28)	0.01 (−0.06, 0.08)		0.01 (−0.03, 0.05)	0.06 (−0.02, 0.14)	0.06 (−0.05, 0.17)	0.06 (−0.10, 0.22)	0.04 (0.00, 0.09)
	1.02, P_z_ = 0.31	0.31, P_z_ = 0.76		0.51, P_z_ = 0.61	1.48, P_z_ = 0.14	1.03, P_z_ = 0.30	0.72, P_z_ = 0.47	1.83, P_z_ = 0.07
SBP	NA	P_h_ < 0.00001, 96%	NA	P_h_ = 0.80, 0%	P_h_ = 0.41, 0%	P_h_ = 0.0002, 85%	P_h_ = 0.0006, 87%	P_h_ < 0.00001, 96%
		−0.11 (−0.27, 0.04)		3.04 (−0.19, 6.26)	0.52 (−0.30, 0.74)	−0.46 (−3.38, −2.46)	−0.14 (−6.61, 6.33)	0.39 (−0.70, 1.49)
		1.42, P_z_ = 0.16		1.85, P_z_ = 0.06	4.57, P_z_ < 0.00001	0.31, P_z_ = 0.76	0.04, P_z_ = 0.97	0.70, P_z_ = 0.48
DBP	NA	P_h_ < 0.00001, 96%	NA	P_h_ = 0.82, 0%	P_h_ = 0.10, 57%	P_h_ = 0.001, 81%	P_h_ = 0.0009, 86%	P_h_ < 0.00001, 97%
		−0.79 (−0.90, −0.68)		2.72 (0.37, 5.07)	0.30 (−0.99, 1.59)	−0.68 (−2.32, 0.96)	0.54 (−3.68, 4.77)	−0.40 (−1.28, 0.48)
		14.13, P_z_ < 0.00001		2.27, P_z_ = 0.02	0.45, P_z_ = 0.65	0.81, P_z_ = 0.42	0.25, P_z_ = 0.80	0.90, P_z_ = 0.37
BMI	P_h_ = 0.002, 69%	P_h_ = 1.00, 0%	NA	NA	P_h_ = 0.06, 72%	P_h_ < 0.00001, 80%	P_h_ = 0.002, 69%	P_h_ < 0.00001, 91%
	−2.04 (−2.49, −1.58)	−0.10 (−0.66, 0.46)			−0.96 (−3.06, 1.15)	−1.82 (−2.32, −1.33)	−2.06 (−2.57, −1.56)	−0.68 (−1.93, 0.56)
	8.74, P_z_ < 0.00001	Z = 0.35, P_z_ = 0.72			0.89, P_z_ = 0.37	7.24, P_z_ < 0.00001	8.00, P_z_ < 0.00001	1.07, P_z_ = 0.28
BW	P_h_ = 0.03, 72%	P_h_ = 0.96, 0%	NA	P_h_ = 0.54, 0%	P_h_ = 0.93, 0%	P_h_ < 0.00001, 96%	P_h_ = 0.44, 0%	P_h_ < 0.00001, 96%
	−7.01 (−9.97, −4.04)	0.35 (−1.71, 2.40)		0.30 (−0.14, 0.73)	0.42 (−0.17, 1.02)	−2.71 (−4.97, −0.45)	−8.76 (−11.18, −6.34)	−0.86 (−3.12, 1.40)
	4.53, P_z_ < 0.00001	0.33, P_z_ = 0.74		1.35, P_z_ = 0.18	1.40, P_z_ = 0.16	2.35, P_z_ = 0.02	7.09, P_z_ < 0.00001	0.75, P_z_ = 0.45

P_h_, *p*-values for heterogeneity of Q-test; MD, mean difference; CI, confidence interval; P_z_, *p*-values for Z-test; P_Z_ < 0.05, shows a significant difference; PEG-Loxe, polyethylene glycol loxenatide; CHD, conventional hypoglycemic drugs; PEG-Loxe + CHD, polyethylene glycol loxenatide combined with conventional hypoglycemic drugs; CHD + Placebo, conventional hypoglycemic drugs combined with Placebo; HbA1c, glycosylated hemoglobin; FPG, fasting plasma glucose; 2-h PBG, 2-h postprandial blood glucose; TC, total cholesterol; TG, triglycerides; LDL-C, low-density lipoprotein cholesterol; HDL-C, high-density lipoprotein cholesterol; SBP, systolic blood pressure; DBP, diastolic blood pressure; BMI, body mass index; BW, body weight; NA, not available.

#### 3.2.1 Meta-analysis of BG: HbA1c; FPG; 2-h PBG

**FIGURE 3:**
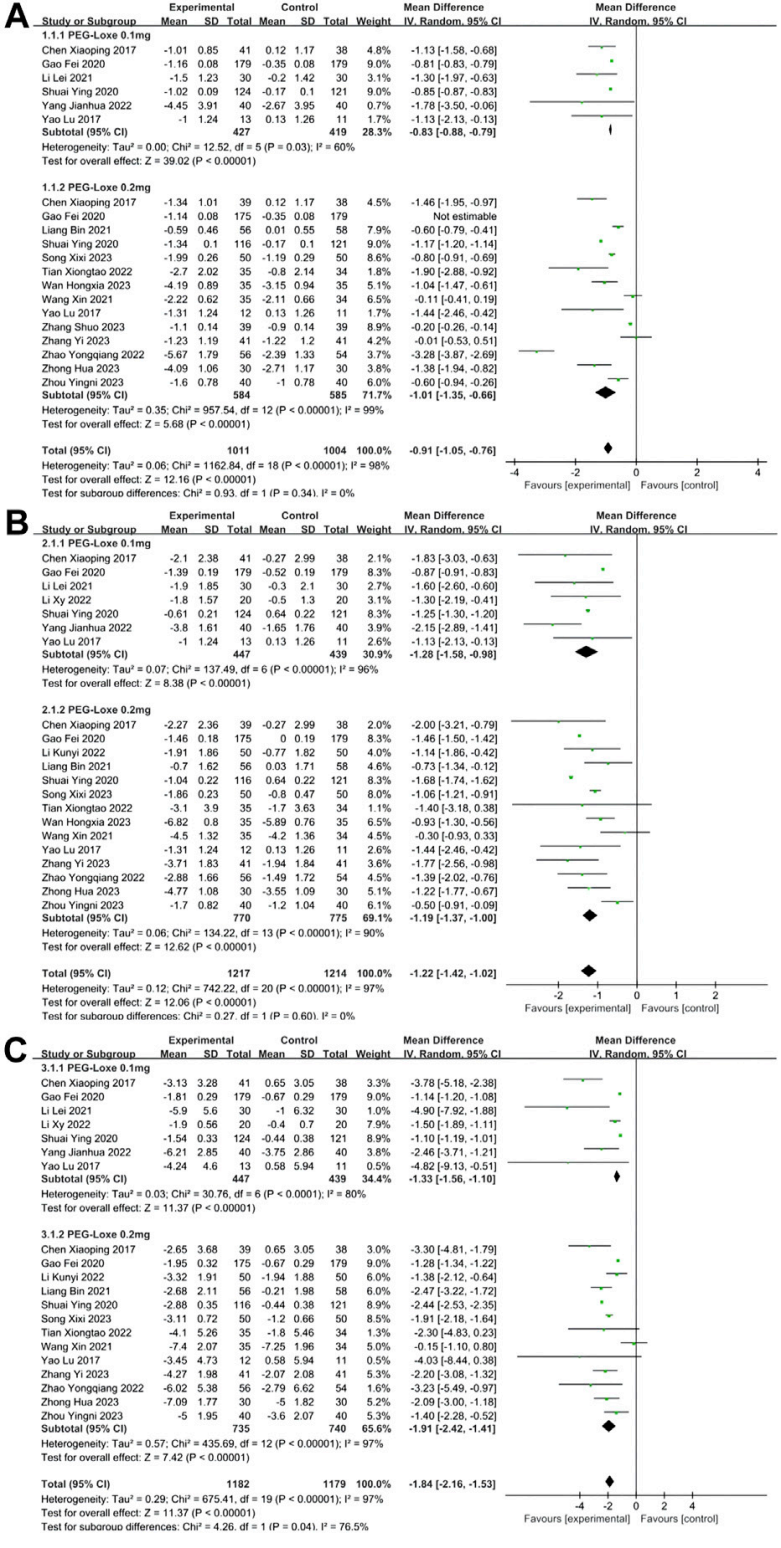
The forest plot of meta-analysis of blood glucose. **(A)** The forest plot of meta-analysis of HbA1c. **(B)** The forest plot of meta-analysis of FPG. **(C)** The forest plot of meta-analysis of 2-h PBG.

HbA1c was reported in 16 studies (Chen et al., 2017; Yao et al., 2017; Gao et al., 2020; Li et al., 2021; Liang et al., 2021; Shuai et al., 2021; Wang and Zhao, 2021; Tian et al., 2022; Yang, 2022; Zhao et al., 2022; Song et al., 2023; Wan et al., 2023; Zhang S. et al., 2023; Zhang Y. et al., 2023; Zhong, 2023; Zhou et al., 2023), whereas FPG and 2-h PBG were reported in 17 (Chen et al., 2017; Yao et al., 2017; Gao et al., 2020; Li et al., 2021; Liang et al., 2021; Shuai et al., 2021; Wang and Zhao, 2021; Li K. et al., 2022; Li X. Y. et al., 2022; Tian et al., 2022; Yang, 2022; Zhao et al., 2022; Song et al., 2023; Wan et al., 2023; Zhang S. et al., 2023; Zhong, 2023; Zhou et al., 2023) and 16 studies (Chen et al., 2017; Yao et al., 2017; Gao et al., 2020; Li et al., 2021; Liang et al., 2021; Shuai et al., 2021; Wang and Zhao, 2021; Li K. et al., 2022; Li X. Y. et al., 2022; Tian et al., 2022; Yang, 2022; Zhao et al., 2022; Song et al., 2023; Zhang Y. et al., 2023; Zhong, 2023; Zhou et al., 2023), respectively. Meta-analysis showed that PEG-Loxe significantly reduced the levels of HbA1c (MD = −0.91; 95% CI, −1.05 to −0.76; P_Z_<0.00001; I^2^ = 98%), FPG (MD = −1.22; 95% CI, −1.42 to −1.02; P_Z_<0.0001; I^2^ = 97%) and 2-h PBG (MD = −1.84, 95% CI, −2.16 to −1.53; P_Z_<0.00001; I^2^ = 97%) in experimental group compared those in control group (Table 2). The forest plots of meta-analysis of HbA1c, FPG and 2-h PBG are showed in Figures 3A–C, respectively. Results obtained from subgroup analyses are shown in Table 3. In 0.2 mg subgroup, the decreased levels of HbA1c (MD = −1.01; 95% CI, −1.35 to −0.66; P_Z_<0.00001; I^2^ = 99%), FPG (MD = −1.19; 95% CI, −1.37 to −1.00; P_Z_<0.00001; I^2^ = 87%) and 2-h PBG (MD = −1.91; 95% CI, −2.42 to −1.41; P_Z_<0.00001; I^2^ = 97%) were more significant than the levels of HbA1c (MD = −0.83; 95% CI, −0.88 to −0.79; P_Z_ = 0.0001; I^2^ = 60%), FPG (MD = −1.28; 95% CI, −1.58 to −0.98; P_Z_<0.00001; I^2^ = 96%) and 2-h PBG (MD = −1.33; 95%CI, −1.56 to −1.10; P_Z_<0.0001; I^2^ = 80%) in 0.1 mg subgroup. Subgroup analysis indicated that the HbA1c and 2-h PBG lowering effects in PEG-Loxe + CHD group were better than that in CHD group and CHD + Placebo group (P_Z_≤0.05). The glucose-lowering effect in PEG-Loxe group was better than that in Placebo group (P_Z_≤0.00001). Other results were not of statistical difference. In addition, subgroup analysis also showed that the high heterogeneity of HbA1c was caused by intervention measures and dosages, and heterogeneity of FPG was caused by intervention measures and treatment time, and heterogeneity of 2-h PBG was caused by intervention measures and treatment time.

#### 3.2.2 Meta-analysis of BLP: TC; TG; LDL-C; HDL-C

**FIGURE 4:**
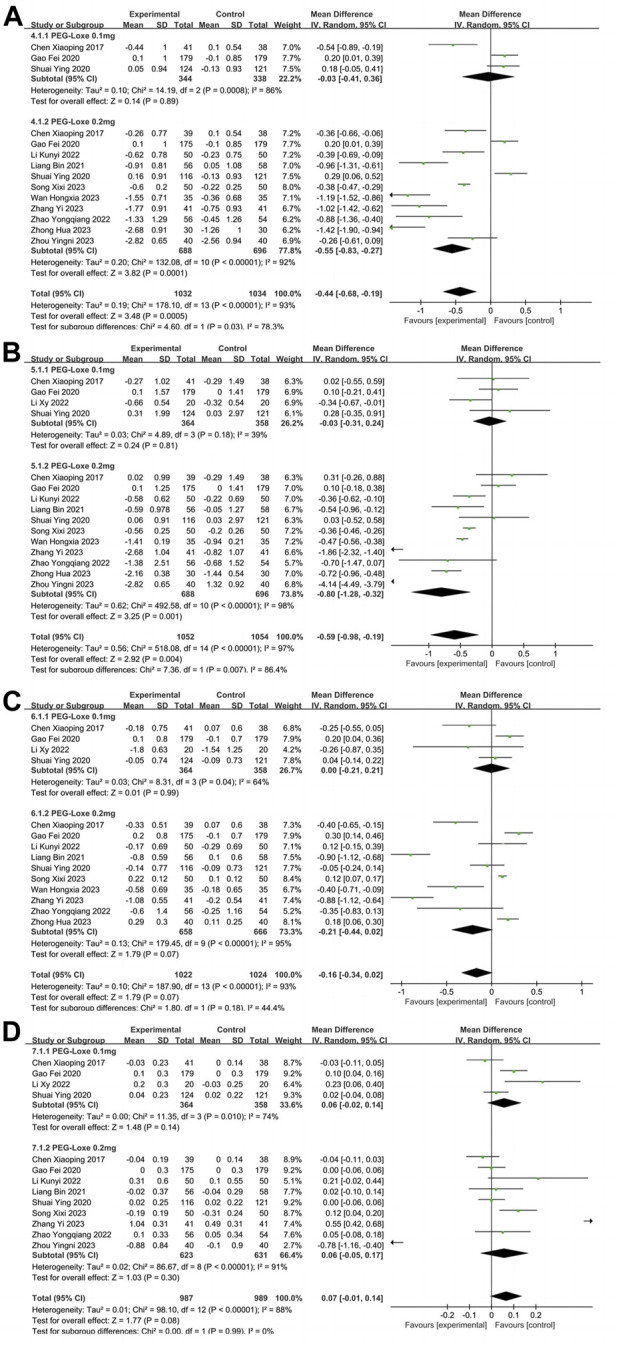
The forest plot of meta-analysis of blood lipid profiles. **(A)** The forest plot of meta-analysis of TC. **(B)** The forest plot of meta-analysis of TG. **(C)** The forest plot of meta-analysis of LDL-C. **(D)** The forest plot of meta-analysis of HDL-C.

TC, TG, LDL-C and HDL-C were reported in 11 (Chen et al., 2017; Gao et al., 2020; Liang et al., 2021; Shuai et al., 2021; Li K. et al., 2022; Zhao et al., 2022; Song et al., 2023; Wan et al., 2023; Zhang S. et al., 2023; Zhong, 2023; Zhou et al., 2023), 12, (Chen et al., 2017; Gao et al., 2020; Liang et al., 2021; Shuai et al., 2021; Li K. et al., 2022; Li X. Y. et al., 2022; Zhao et al., 2022; Song et al., 2023; Wan et al., 2023; Zhang Y. et al., 2023; Zhong, 2023; Zhou et al., 2023), 11 (Chen et al., 2017; Gao et al., 2020; Liang et al., 2021; Shuai et al., 2021; Li K. et al., 2022; Li X. Y. et al., 2022; Zhao et al., 2022; Song et al., 2023; Wan et al., 2023; Zhang S. et al., 2023; Zhou et al., 2023), and 10 (Chen et al., 2017; Gao et al., 2020; Liang et al., 2021; Shuai et al., 2021; Li K. et al., 2022; Li X. Y. et al., 2022; Zhao et al., 2022; Song et al., 2023; Zhang Y. et al., 2023; Zhou et al., 2023) studies, respectively. In Table 2, the overall analysis of BLP showed that changes of TC (MD = −0.44; 95% CI, −0.68 to 0.19; P_Z_ = 0.0005; I^2^ = 93%), TG (MD = −0.59; 95% CI, −0.98 to 0.19; P_Z_ = 0.004; I^2^ = 97%), LDL-C (MD = −0.16; 95% CI, −0.34 to 0.02; P_Z_ = 0.07; I^2^ = 93%), and HDL-C (MD = 0.07; 95% CI, −0.01 to 0.14; P_Z_ = 0.08; I^2^ = 88%) in experimental group were not statistically significant compared with control group. The forest plot of meta-analysis of TC, TG, LDL-C, and HDL-C are shown in Figures 4A–D, respectively. Since the control group was treated with placebo or other CHD, the difference in BLP between the experimental and control groups might have been less significant than what would have been observed in control group with only placebo applied. In Table 3, Intervention measure subgroup analysis showed that the effect of reducing TC and TG of PEG-Loxe + CHD group were better than those in CHD group (P_Z_≤0.00001), and the improvement effect of PEG Loxe on TC did not show any advantage compared with Placebo group (P_Z_ = 0.005). In 0.2 mg subgroup, the decreased levels of TC and TG were significant. Treatment time subgroup showed that the changes in TC at 12 weeks, LDL-C at 12 and 24 weeks were statistically significant. And other results were not of statistical difference. In addition, subgroup analysis also showed that the high heterogeneities of TC, LDL-C, and HDL-C were caused by the intervention measures, and the high heterogeneity of TG was caused by intervention measures and dosages.

#### 3.2.3 Meta-analysis of BP: SBP and DBP

**FIGURE 5:**
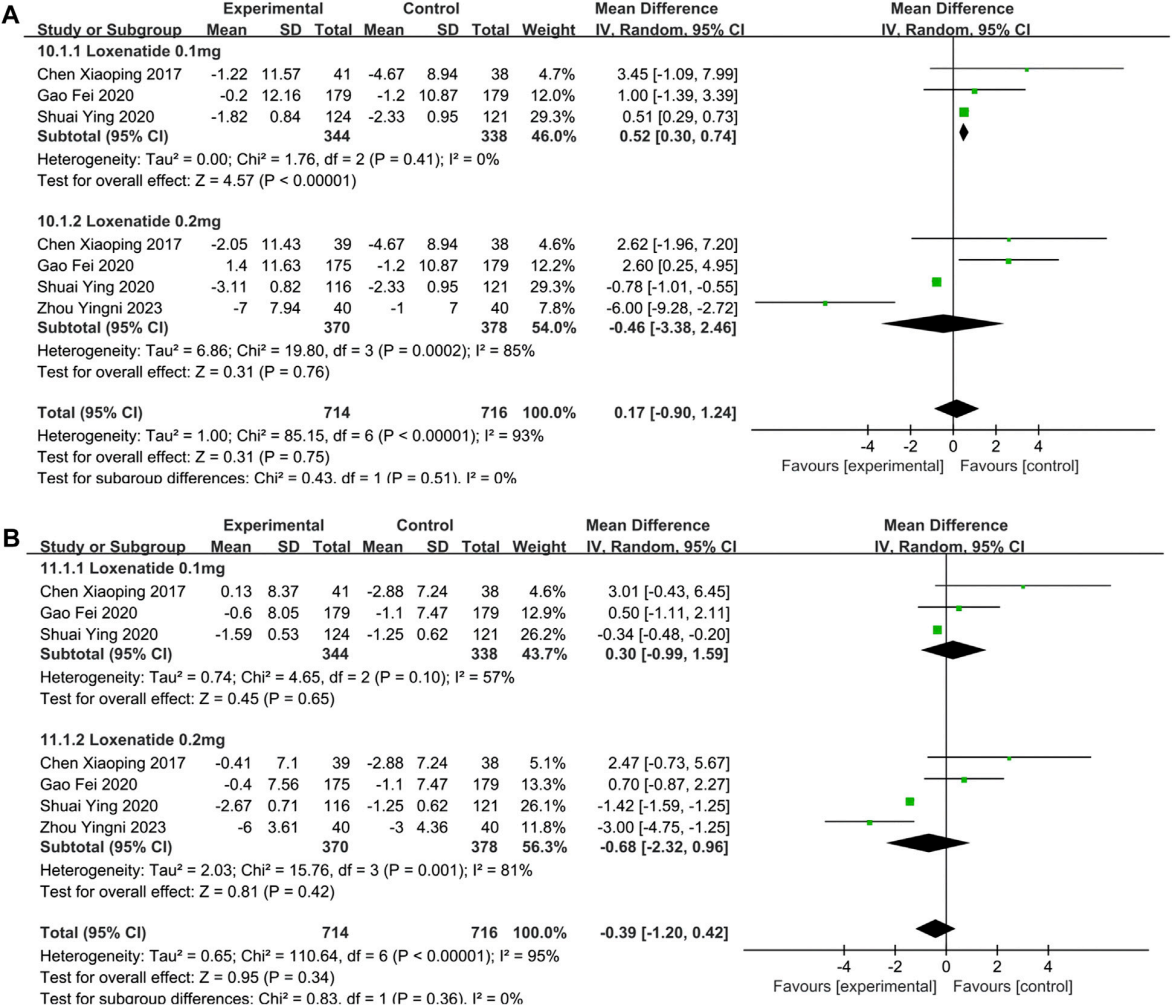
The forest plot of meta-analysis of SBP and DBP. **(A)** The forest plot of meta-analysis of SBP. **(B)** The forest plot of meta-analysis of DBP.

SBP and DBP were reported in 4 studies (Chen et al., 2017; Gao et al., 2020; Shuai et al., 2021; Zhou et al., 2023). In Table 2, the overall analysis of BP showed that changes of SBP (MD = 0.17; 95% CI, −0.90 to 1.24; P_Z_ = 0.75; I^2^ = 93%) and DBP (MD = −0.39; 95% CI, −1.20 to 0.42; P_Z_ = 0.34; I^2^ = 95%) in experimental group were not statistically significant compared with control group. The forest plot of meta-analysis of SBPand DBP are shown in Figures 5A, B, respectively. In Table 3, intervention measure subgroup analysis showed that the effect of reducing DBP of PEG-Loxe + CHD group were better than those in CHD + Placebo group (P_z_ < 0.00001), and the improvement effect of PEG Loxe on DBP did not show any advantage compared with Placebo group (P_Z_ = 0.02). And other groups were not statistically different. In addition, subgroup analysis also showed that the high heterogeneities of SBP and DBP were caused by the intervention measures.

#### 3.2.4 Meta-analysis of BMI and BW

**FIGURE 6:**
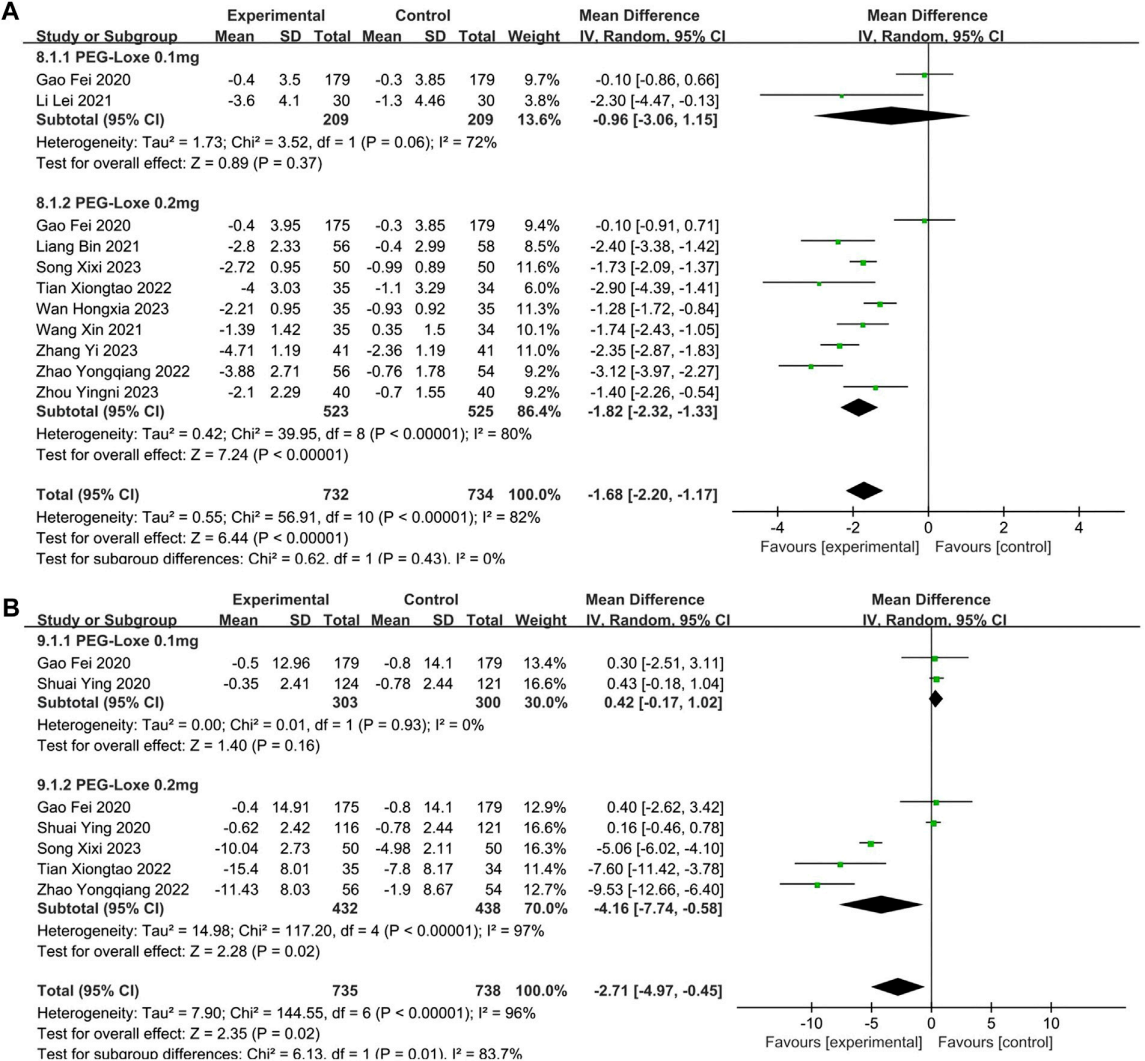
The forest plot of meta-analysis of BMI and BW. **(A)** The forest plot of meta-analysis of BMI. **(B)** The forest plot of meta-analysis of BW.

The changes in BMI and BW were reported by 10 (Gao et al., 2020; Li et al., 2021; Liang et al., 2021; Wang and Zhao, 2021; Tian et al., 2022; Zhao et al., 2022; Song et al., 2023; Wan et al., 2023; Zhang S. et al., 2023; Zhou et al., 2023) and 5 (Gao et al., 2020; Shuai et al., 2021; Tian et al., 2022; Zhao et al., 2022; Song et al., 2023) studies, respectively. Significant reductions in BMI (MD = −1.68; 95% CI, −2.20 to −1.17; P_Z_ < 0.00001; I^2^ = 82%) and BW (MD = −2.71; 95% CI, −4.97 to −0.45; P_Z_ = 0.02; I^2^ = 96%) are shown in Table 2. The forest plot of meta-analysis of BMI and BW are shown in Figures 6A, B, respectively. In Table 3, subgroup analyses on intervention measures showed that the effect of reducing BMI and BW in PEG-Loxe + CHD group were better than that in CHD group (P_Z_ < 0.0001), 0.2 mg PEG-Loxe caused a statistically significant change in BMI (P_Z_ < 0.00001) and BW (P_Z_ = 0.004) in dosages subgroup. In treatment time subgroup, PEG-Loxe caused statistically significant changes in BMI and BW (P_Z_ < 0.00001) at 12 weeks, while other results were not of statistical difference. In addition, subgroup analysis also showed that the high heterogeneity of BMI was caused by intervention measures, and the high heterogeneity of BW was intervention measures, dosage and treatment time.

## 4 Sensitivity analysis

Sensitivity analysis was performed on the combined results of the indicators. The results of the meta-analysis were considered robust as there was no significant change in the combined effect size after removing a trial at a time.

## 5 Discussion

This is the first study to systematically assess the effects of PEG-Loxe on BG, BLP, BP, BMI, and BW. The overall results showed that PEG-Loxe was significantly effective in reducing HbA1c, FPG, 2-h PBG, BMI, and BW in patients with T2DM, but was not effective for improving TC, TG, HDL-C, LDL-C, SBP, and DBP. This suggested that PEG-Loxe might have a significant effect on lowering BG and reducing BW. We divided participants into subgroups based on different intervention measures, dosages, and treatment time. Then a comprehensive subgroup analysis was performed according to different variables to explain or explore the sources of heterogeneity. The above variables were identified as the source of high heterogeneity in the research results through subgroup analysis. In addition, the results of subgroup analysis showed that PEG-Loxe combined with CHD showed better effects in reducing HbA1c, FPG, 2-h PBG, TC, TG, BMI, and BW compared with CHD. And PEG-Loxe showed better hypoglycemic effects than placebo. In each subgroup, the heterogeneity of the results was greatly reduced.

Chronic hyperglycemia was the most typical pathologic manifestation of T2DM. Hyperglycemia increased the urine output of patients, which could lead to electrolyte disorders, hypertonic diuresis, and dehydration of the body (Fayfman et al., 2017; Sun et al., 2020). Also, hyperglycemia could cause diabetic nephropathy in patients with T2DM. The early symptoms of diabetic nephropathy are proteinuria and edema, while the late stage is renal failure that was the main cause of death in T2DM (Samsu, 2021). Hyperglycemia state could lead to excessive breakdown of fat and protein, and further secondary infections, such as boils of skin, wound infection, lung infection, and urinary tract infection (Nagendra et al., 2000). Long term of hyperglycemia has a toxic effect on the pancreatic islet beta-cells, and would accelerate the pancreatic islet beta-cells apoptosis and pancreatic islet failure, leading to gradual deterioration of the condition (Eizirik et al., 2020). In addition, long term hyperglycemia in diabetic patients would damage large vessels and micro-vessels, and sensory nerves and autonomic nerves, which would cause the occurrence and development of chronic complications such as cardiovascular and cerebrovascular diseases, diabetes nephropathy, retinopathy, peripheral neuropathy, diabetes foot gangrene (Jia et al., 2018; Eckel et al., 2021). Overweight and obesity are risk factors for cardiovascular disease, and it can affect cardiovascular health by influencing metabolic syndromes such as insulin resistance and dyslipidemia (Kachur et al., 2017; Che et al., 2018). Therefore, it could be concluded that control of BG and BW is important in the treatment of T2DM (Davies et al., 2022). The weight loss effect of PEG-Loxe may inhibit the development of T2DM patients to T2DM complicated with cardiovascular disease.

PEG-Loxe reduces HbA1c in a similar manner to other GLP-1RAs. More importantly, it is the only GLP-1RA that increases the therapeutic dosage without increasing the risk of hypoglycemia (Jiang et al., 2021). Therefore, PEG-Loxe has multiple therapeutic advantages. In terms of mechanism of action, PEG-Loxe improves beta-cells function and plays a hypoglycemic role by stimulating insulin secretion, inhibiting glucagon secretion, improving insulin resistance, and inhibiting hepatic glucose output by activating insulin phosphatidyl inositol 3-kinase/protein kinase B (PI3K/AKT) pathway (Rameshrad et al., 2020; Ard et al., 2021; Zeng et al., 2021; Zhang et al., 2021). Other studies have found that PEG-Loxe could regulate the expression of chemerin and omentin through its hypoglycemic effect (Li X. Y. et al., 2022). In addition, PEG-Loxe can delay gastric emptying and suppress patients’ appetite, thereby reducing their food intake and ultimately reducing their weight (Drucker et al., 2017). And studies have reported that PEG-Loxe could regulating gut microbiota to protect vascular endothelial cell function in T2DM patients (Chen et al., 2022). Since there are few studies on the mechanism of PEG-Loxe, further studies are needed to prove its specific pharmacological mechanism.

There exist a couple of limitations in the research. Firstly, meta-analysis results showed some heterogeneity. We found that intervention measures, dosages and course of treatment were the causes of high heterogeneity by subgroup analysis. Secondly, since the control group was treated with a placebo or other hypoglycemic agents, the difference of meta-analysis in BLP between the experimental and control groups might have been less significant than what would have been observed in the control group with only placebo applied. And in some studies, BLP were not the primary endpoint, so enrolled patients may not have dyslipidemia, which may be why no difference in BLP was observed. Thirdly, since PEG-Loxe is a novel drug, meta-analysis was limited by sample sizes and a short study period, and its long-term efficacy cannot be evaluated temporarily, longer duration of observation is need in further. Besides, PEG-Loxe is independently developed in China, and correspondingly 11 of the 18 studies included were published in Chinese journals, and the conclusions of the meta-analysis may be more applicable for East Asian. And SBP and DBP indicators were reported in 4 studies only, the results of its meta-analysis need to be viewed with caution. In the future, more high-quality, large-sample, multicenter RCTs of PEG-Loxe for T2DM should be performed.

In summary, PEG-Loxe is a promising drug in controlling BG and BW for patients with T2DM, and is worthy of promoting in clinical practice. In the future, more high-quality, large-sample, multicenter RCTs should be conducted to explore its impact on blood lipids further and provide a more rational basis and reference for treating T2DM clinically.

## 6 Conclusion

PEG-Loxe has better hypoglycemic effects compared with placebo in patients with T2DM, but could not significantly improved TC, TG, LDL-C, HDL-C, SBP, DBP and BW. And the combination of CHD and PEG-Loxe could more effectively improve the levels of HbA1c, FPG, 2-h PBG, TC, TG, BMI, and BW compared with CHD in T2DM patients.

## Data Availability

The original contributions presented in the study are included in the article/Supplementary Material, further inquiries can be directed to the corresponding authors.
