# Supplementary material for: Efficacy of polyethylene glycol loxenatide for type 2 diabetes mellitus patients: a systematic review and meta-analysis
Source: Front Pharmacol. 2024 Feb 26;15:1235639. doi: 10.3389/fphar.2024.1235639 (PMC10925615; doi:10.3389/fphar.2024.1235639)
Supplement: Supplementary file 1 [file Table1.DOC]

Table S1.The Search strategy in this study.

| Literature databases | Search items |
| --- | --- |
| Pubmed | ((((("Diabetes Mellitus, Type 2"[Mesh]) OR (（Diabetes Mellitus, Noninsulin-Dependent[Title/Abstract]) OR（Diabetes Mellitus, Ketosis-Resistant[Title/Abstract]) OR（Diabetes Mellitus, Ketosis Resistant[Title/Abstract]) OR（Ketosis-Resistant Diabetes Mellitus[Title/Abstract]) OR（Diabetes Mellitus, Non Insulin Dependent[Title/Abstract]) OR（Diabetes Mellitus, Non-Insulin-Dependent[Title/Abstract]) OR（Non-Insulin-Dependent Diabetes Mellitus[Title/Abstract]) OR（Diabetes Mellitus, Stable[Title/Abstract]) OR（Stable Diabetes Mellitus[Title/Abstract]) OR（Diabetes Mellitus, Type II[Title/Abstract]) OR（NIDDM[Title/Abstract])OR（Diabetes Mellitus, Noninsulin Dependent[Title/Abstract]) OR（Diabetes Mellitus, Maturity-Onset[Title/Abstract]) OR（Diabetes Mellitus, Maturity Onset[Title/Abstract]) OR（Maturity-Onset Diabetes Mellitus[Title/Abstract]) OR（Maturity Onset Diabetes Mellitus[Title/Abstract]) OR（MODY[Title/Abstract]) OR（Diabetes Mellitus, Slow-Onset[Title/Abstract]) OR（Diabetes Mellitus, Slow Onset[Title/Abstract]) OR（Slow-Onset Diabetes Mellitus[Title/Abstract] )OR（Type 2 Diabetes Mellitus[Title/Abstract]) OR（Noninsulin-Dependent Diabetes Mellitus[Title/Abstract]) OR（Noninsulin Dependent Diabetes Mellitus[Title/Abstract]) OR（Maturity-Onset Diabetes[Title/Abstract]) OR（Diabetes, Maturity-Onset[Title/Abstract]) OR（Maturity Onset Diabetes[Title/Abstract]) OR（Type 2 Diabetes[Title/Abstract]) OR（Diabetes, Type 2[Title/Abstract]) OR（Diabetes Mellitus, Adult-Onset[Title/Abstract]) OR（Adult-Onset Diabetes Mellitus[Title/Abstract]) OR（Diabetes Mellitus, Adult Onset[Title/Abstract])))) AND (("polyethylene glycol loxenatide" [Supplementary Concept]) OR ((PEX168) OR (PEG-Loxe)))) AND ((randomized controlled trial[Publication Type]) OR ((randomized) OR (placebo)))) |
| Cochrane Library | (Diabetes Mellitus, Type 2):ti,ab,kw OR (Diabetes Mellitus, Noninsulin-Dependent):ti,ab,kw OR (Diabetes Mellitus, Ketosis-Resistant):ti,ab,kw OR (Diabetes Mellitus, Ketosis Resistant):ti,ab,kw OR(Ketosis-Resistant Diabetes Mellitus):ti,ab,kw OR (Diabetes Mellitus, Non Insulin Dependent):ti,ab,kw OR (Diabetes Mellitus, Non-Insulin-Dependent):ti,ab,kw OR (Non-Insulin-Dependent Diabetes Mellitus):ti,ab,kw OR (Diabetes Mellitus, Stable):ti,ab,kw OR (Stable Diabetes Mellitus):ti,ab,kw OR (Diabetes Mellitus, Type II):ti,ab,kw OR (NIDDM):ti,ab,kw OR(Diabetes Mellitus, Noninsulin Dependent):ti,ab,kw OR (Diabetes Mellitus, Maturity-Onset):ti,ab,kw OR (Diabetes Mellitus, Maturity Onset):ti,ab,kw OR (Maturity-Onset Diabetes Mellitus):ti,ab,kw OR (Maturity Onset Diabetes Mellitus):ti,ab,kw OR (MODY):ti,ab,kw OR(Diabetes Mellitus, Slow-Onset):ti,ab,kw OR (Diabetes Mellitus, Slow Onset):ti,ab,kw OR (Slow-Onset Diabetes Mellitus):ti,ab,kw OR (Type 2 Diabetes Mellitus):ti,ab,kw OR (Noninsulin-Dependent Diabetes Mellitus):ti,ab,kw OR (Noninsulin Dependent Diabetes Mellitus):ti,ab,kw OR (Maturity-Onset Diabetes):ti,ab,kw OR (Diabetes, Maturity-Onset):ti,ab,kw OR (Maturity Onset Diabetes):ti,ab,kw OR(Type 2 Diabetes):ti,ab,kw OR (Diabetes, Type 2):ti,ab,kw OR (Diabetes Mellitus, Adult-Onset):ti,ab,kw OR (Adult-Onset Diabetes Mellitus):ti,ab,kw OR (Diabetes Mellitus, Adult Onset):ti,ab,kw AND (polyethylene glycol loxenatide):ti,ab,kw OR (PEX168):ti,ab,kw AND (randomized controlled trial):ti,ab,kw OR (randomized):ti,ab,kw OR (placebo):ti,ab,kw |
| Embase | 'Diabetes Mellitus, Noninsulin-Dependent'/exp or 'Diabetes Mellitus, Ketosis-Resistant':ab,ti or 'Diabetes Mellitus, Ketosis Resistant':ab,ti or 'Ketosis-Resistant Diabetes Mellitus':ab,ti or 'Diabetes Mellitus, Non Insulin Dependent':ab,ti or 'Diabetes Mellitus, Non-Insulin-Dependent':ab,ti or 'Non-Insulin-Dependent Diabetes Mellitus':ab,ti or 'Diabetes Mellitus, Stable':ab,ti or 'Stable Diabetes Mellitus':ab,ti or 'Diabetes Mellitus, Type II':ab,ti or 'NIDDM':ab,ti or 'Diabetes Mellitus, Noninsulin Dependent':ab,ti or 'Diabetes Mellitus, Maturity-Onset':ab,ti or 'Diabetes Mellitus, Maturity Onset':ab,ti or 'Maturity-Onset Diabetes Mellitus':ab,ti or 'Maturity Onset Diabetes Mellitus':ab,ti or 'MODY':ab,ti or 'Diabetes Mellitus, Slow-Onset':ab,ti or 'Diabetes Mellitus, Slow Onset':ab,ti or 'Slow-Onset Diabetes Mellitus':ab,ti or 'Type 2 Diabetes Mellitus':ab,ti or 'Noninsulin-Dependent Diabetes Mellitus':ab,ti or 'Noninsulin Dependent Diabetes Mellitus':ab,ti or 'Maturity-Onset Diabetes':ab,ti or 'Diabetes, Maturity-Onset':ab,ti or 'Maturity Onset Diabetes':ab,ti or 'Type 2 Diabetes':ab,ti or 'Diabetes, Type 2':ab,ti or 'Diabetes Mellitus, Adult-Onset':ab,ti or 'Adult-Onset Diabetes Mellitus':ab,ti or 'Diabetes Mellitus, Adult Onset':ab,ti AND 'polyethylene glycol loxenatide':ab,ti or 'PEX168':ab,ti or 'PEX168':ab,ti AND 'randomized controlled trial':ab,ti or 'randomized':ab,ti or 'placebo' :ab,ti |
| Web of Science | TS=(Ketosis-Resistant Diabetes Mellitus OR Non-Insulin-Dependent Diabetes Mellitus OR Stable Diabetes Mellitus OR NIDDM OR Maturity-Onset Diabetes Mellitus OR Maturity Onset Diabetes Mellitus OR MODY OR Slow-Onset Diabetes Mellitus OR Type 2 Diabetes Mellitus OR Noninsulin-Dependent Diabetes Mellitus OR Noninsulin Dependent Diabetes Mellitus OR Maturity-Onset Diabetes OR Maturity Onset Diabetes OR Type 2 Diabetes OR Adult-Onset Diabetes Mellitus) AND TS=(polyethylene glycol loxenatide OR PEX168 OR PEG-Loxe) AND TS=(randomized controlled trial OR randomized OR placebo) |
| Medline | TS=(Ketosis-Resistant Diabetes Mellitus OR Non-Insulin-Dependent Diabetes Mellitus OR Stable Diabetes Mellitus OR NIDDM OR Maturity-Onset Diabetes Mellitus OR Maturity Onset Diabetes Mellitus OR MODY OR Slow-Onset Diabetes Mellitus OR Type 2 Diabetes Mellitus OR Noninsulin-Dependent Diabetes Mellitus OR Noninsulin Dependent Diabetes Mellitus OR Maturity-Onset Diabetes OR Maturity Onset Diabetes OR Type 2 Diabetes OR Adult-Onset Diabetes Mellitus) AND TS=(polyethylene glycol loxenatide OR PEX168 OR PEG-Loxe) AND TS=(randomized controlled trial OR randomized OR placebo) |
| Scopus | TITLE-ABS-KEY("Diabetes Mellitus, Type 2") OR TITLE-ABS-KEY("Diabetes Mellitus, Noninsulin-Dependent") OR TITLE-ABS-KEY("Diabetes Mellitus, Ketosis-Resistant") OR TITLE-ABS-KEY("Diabetes Mellitus, Ketosis Resistant") OR TITLE-ABS-KEY("Ketosis-Resistant Diabetes Mellitus") OR TITLE-ABS-KEY("Diabetes Mellitus, Non Insulin Dependent") OR TITLE-ABS-KEY("Diabetes Mellitus, Non-Insulin-Dependent") OR TITLE-ABS-KEY("Non-Insulin-Dependent Diabetes Mellitus") OR TITLE-ABS-KEY("Diabetes Mellitus, Stable") OR TITLE-ABS-KEY("Stable Diabetes Mellitus") OR TITLE-ABS-KEY("Diabetes Mellitus, TITLE-ABS-KEY("") II") OR TITLE-ABS-KEY("NIDDM") OR TITLE-ABS-KEY("Diabetes Mellitus, Noninsulin Dependent") OR TITLE-ABS-KEY("Diabetes Mellitus, Maturity-Onset") OR TITLE-ABS-KEY("Diabetes Mellitus, Maturity Onset") OR TITLE-ABS-KEY("Maturity-Onset Diabetes Mellitus") OR TITLE-ABS-KEY("Maturity Onset Diabetes Mellitus") OR TITLE-ABS-KEY("MODY") OR TITLE-ABS-KEY("Diabetes Mellitus, Slow-Onset") OR TITLE-ABS-KEY("Diabetes Mellitus, Slow Onset") OR TITLE-ABS-KEY("Slow-Onset Diabetes Mellitus") OR TITLE-ABS-KEY("Type 2 Diabetes Mellitus") OR TITLE-ABS-KEY("Noninsulin-Dependent Diabetes Mellitus") OR TITLE-ABS-KEY("Noninsulin Dependent Diabetes Mellitus") OR TITLE-ABS-KEY("Maturity-Onset Diabetes") OR TITLE-ABS-KEY("Diabetes, Maturity-Onset") OR TITLE-ABS-KEY("Maturity Onset Diabetes") OR TITLE-ABS-KEY("Type 2 Diabetes") OR TITLE-ABS-KEY("Diabetes, Type 2") OR TITLE-ABS-KEY("Diabetes Mellitus, Adult-Onset") OR TITLE-ABS-KEY("Adult-Onset Diabetes Mellitus") OR TITLE-ABS-KEY("Diabetes Mellitus, Adult Onset") AND TITLE-ABS-KEY("polyethylene glycol loxenatide") OR TITLE-ABS-KEY("PEX168") OR TITLE-ABS-KEY("PEG-Loxe") AND TITLE-ABS-KEY("randomized controlled trial") OR TITLE-ABS-KEY("randomized") OR TITLE-ABS-KEY("placebo") |
